# Cognitive function in the context of pharmacogenetic CYP2D6 variability and anticholinergic burden in older adults – results from the ActiFE study

**DOI:** 10.1007/s00228-026-04155-y

**Published:** 2026-08-04

**Authors:** Linda Lorenz, Judith Berres, Michael D Denkinger, Dietrich Rothenbacher, Stefanie Braig, Dhayana Dallmeier, Katja S Just

**Affiliations:** 1 Institute of Clinical Pharmacology, University Hospital RWTH Aachen, Aachen, Germany; 2 Institute for Geriatric Research, Ulm University Medical Centre, Ulm, Germany; 3 Geriatric Centre Ulm, AGAPLESION Bethesda Hospital, Ulm, Germany; 4 Institute of Epidemiology and Medical Biometry, Ulm University, Ulm, Germany; 5 Department of Epidemiology, Boston University School of Public Health, Boston, USA

**Keywords:** Older adults, Anticholinergic burden, CYP2D6, Pharmacogenetics, Cognitive impairment

## Abstract

**Purpose:**

To investigate the role of CYP2D6 and its pharmacogenetic variability in the association between anticholinergic burden and cognitive function in older adults.

**Methods:**

We conducted a cross-sectional analysis of 872 community-dwelling adults ≥ 65 years from the ActiFE-Ulm cohort. Cognitive performance was assessed using the Mini-Mental State Examination (MMSE). Anticholinergic burden scores deriving from CYP2D6-metabolised drugs (ABS_2D6_) were calculated for all regularly scheduled medications. CYP2D6 metaboliser status was determined via genotyping and categorised as poor (PM), intermediate (IM), normal (NM), or ultrarapid (UM). The 25th, 50th, and 75th percentiles of the MMSE score distribution were studied using quantile regression to examine the association between ABS_2D6_ and cognition, including interaction and stratified analyses by metaboliser status.

**Results:**

ABS_2D6_ was significantly associated with lower MMSE scores at the 50th and 75th percentiles in early models, but not after additional adjustment for age and sex. A consistent effect modification by IM status was observed at the 75th percentile, with IMs showing a significant negative association between ABS_2D6_ and MMSE in stratified analyses (β = -0.34 [95% CI: -0.64, -0.05]). After excluding participants with use of CYP2D6 inhibitors, this effect became more pronounced (β = -0.50 [95% CI: -0.82, -0.18]). Findings among PMs remained uncertain due to limited subgroup size.

**Conclusion:**

These exploratory findings suggest that CYP2D6 metaboliser status may contribute to interindividual susceptibility to anticholinergic burden-related lower cognitive performance. If confirmed in larger longitudinal studies, pharmacogenetic information may support more individualised anticholinergic risk assessment and safer prescribing in older adults.

**Supplementary Information:**

The online version contains supplementary material available at 10.1007/s00228-026-04155-y.

## Introduction

Between 2025 and 2050, the global number of people aged 60 years and older is projected to nearly double – from approximately 1.2 billion to 2.1 billion – corresponding to an increase from approximately 15% to 22% of the world’s total population [1]. The demographic shift towards an ageing population comes with an increased prevalence of age-related conditions, such as dementia and cognitive impairment [2]. Despite increasing evidence of adverse events, older adults are frequently prescribed anticholinergic drugs [3–6]. Nearly a quarter of the population of adults aged 65 years and older with dementia uses anticholinergic medication, according to a recent systematic review [7]. Anticholinergic drugs block muscarinic cholinergic receptors, thereby inhibiting the effect of acetylcholine, and are used to treat various conditions, including psychiatric, urinary tract-related, and gastrointestinal disorders. The anticholinergic burden, resulting from the use of these drugs, is a known risk factor for a variety of adverse drug reactions (ADRs), ranging from peripheral to central symptoms. Typical peripheral effects – such as dry mouth, urinary retention, and tachycardia – may be easier to detect in clinical everyday practice due to their physical nature. In contrast, central effects – such as impaired concentration, confusion, and attentional deficits – may be less apparent, particularly in older adults with multiple comorbidities [8, 9]. Several studies have reported an association between anticholinergic burden and poorer cognitive performance, cognitive impairment, or cognitive decline in older adults [10–14]. This may be due to the importance of cholinergic pathways for cognition, which are disrupted by anticholinergic medication use [15, 16].

However, cognitive function in older adults is known to be influenced by multiple factors beyond medication use. Risk factors for poorer cognitive function include low educational attainment, vascular comorbidities (e.g., diabetes, hypertension), sensory impairments (e.g., vision and hearing impairment), psychosocial factors such as loneliness and depression, as well as smoking and obesity [17–19]. In addition to these risk factors, pharmacokinetic aspects may further contribute to the extent of anticholinergic effects on cognition. Some anticholinergic drugs are metabolised by drug-metabolising cytochrome P450 (CYP) enzymes. Among them, CYP2D6 is particularly important, as it is involved in the metabolism of approximately 25% of clinically used drugs [20]. CYP2D6 is highly polymorphic, leading to considerable interindividual variation in the metabolism of its substrates and, consequently, differences in drug response and the occurrence of ADRs [21]. Pharmacogenetic (PGx) variants in the CYP2D6 enzyme result in four different metaboliser phenotype groups: poor (PMs), intermediate (IMs), normal (NMs), and ultrarapid metabolisers (UMs) [22]. Although CYP2D6 phenotypes can be predicted by genotyping, non-genetic factors such as drug-drug interactions, health conditions, or environmental impacts can alter the actual capacity to metabolise drugs [23]. This phenomenon, known as phenoconversion, can lead to a conversion of CYP2D6 NMs to CYP2D6 PMs [24].

Serum anticholinergic activity (SAA) levels in nursing home patients with high anticholinergic drug burden were observed to be significantly higher in those being CYP2D6 or CYP2C19 PMs compared to those with functional metabolism [25]. However, no other studies have yet explored the role of the polymorphic CYP2D6 pathway and its metabolic variability in the association between anticholinergic burden and cognitive function.

Thus, the aim of this study was to analyse the anticholinergic burden influenced by the CYP2D6 pathway and its pharmacogenetic variability using cognitive performance as an outcome. We hypothesised that anticholinergic burden deriving from drugs metabolised by CYP2D6 may be associated with cognitive impairment, and that this association would be most pronounced among individuals with genetically reduced CYP2D6 activity.

## Methods

### Study design and participants

**Fig. 1:**
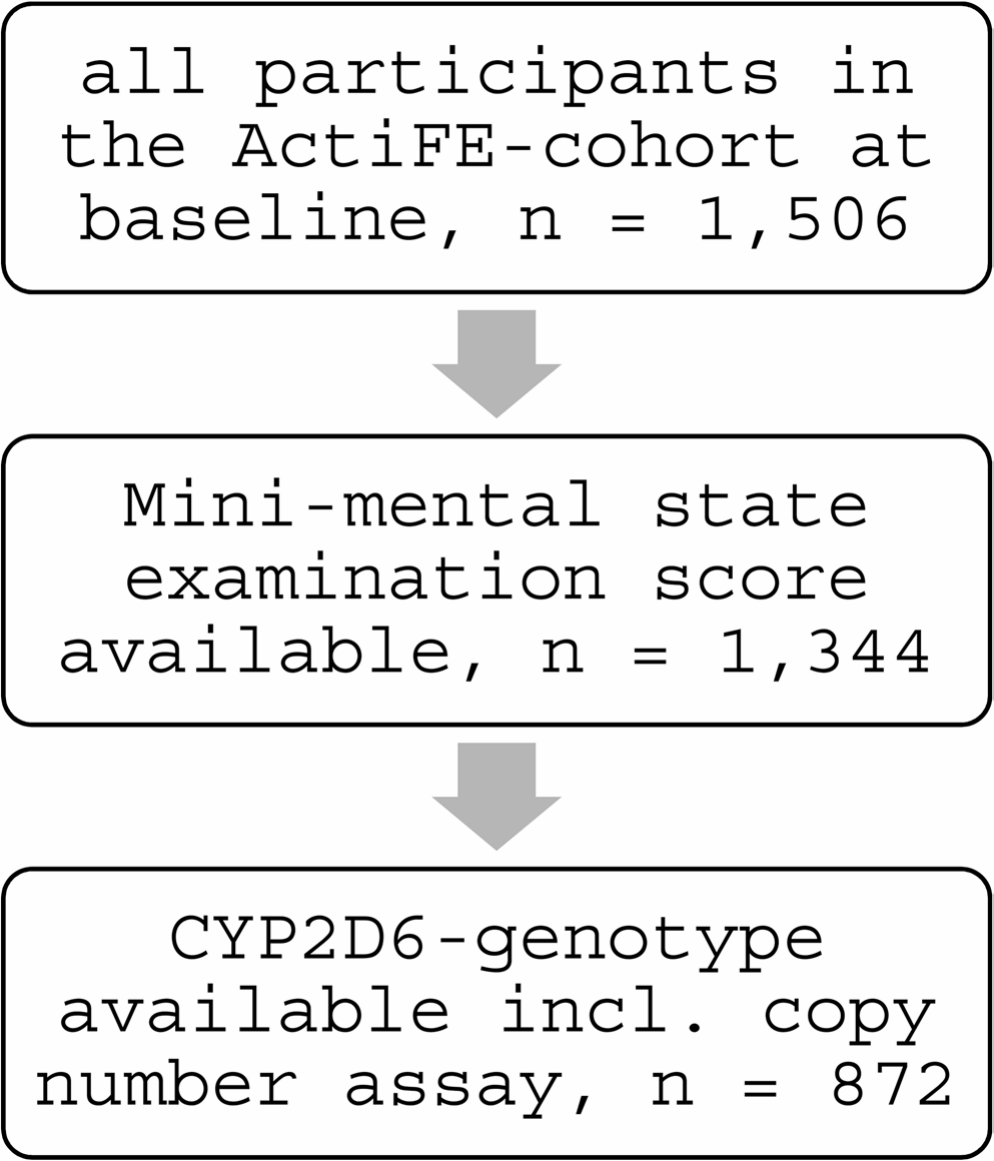
Flow chart of case selection leading to the final study sample for analysis

The cross-sectional analysis is grounded on the baseline assessments of the ActiFE-Ulm study (Activity and Function in the Elderly in Ulm), a prospective population-based study in southern Germany. Details of the study design have been published elsewhere [26]. Non-institutionalised older adults aged between 65 and 90 years who were able to walk through their own room, using assistive devices, if necessary, were eligible for participation. A random sample of 7624 residents from the registration office in Ulm, encompassing the city of Ulm and adjacent regions, was contacted by post and invited to participate. Between March 2009 and April 2010, 1506 eligible individuals agreed to participate (participation rate: 19.8%) and underwent baseline testing. Exclusion criteria were severe visual, hearing, or cognitive impairment, and serious difficulties with the German language. For our analysis, we only included participants with available information on Mini-Mental State Examination (MMSE) score and CYP2D6 genotyping, resulting in a final study sample of 872 participants. Details of the sample selection are presented in Fig. 1.

All participants gave written consent. The ethics committee of Ulm University approved the study (application no. 318/08).

### Data collection

During baseline assessments, the participants completed interviews on sociodemographic characteristics and medical history, including level of education, body mass index (BMI), the history of diabetes and hypertension, using standardised questionnaires. Near vision was measured using the Snellen eye chart (tumbling E’s). Hearing was subjectively evaluated by the interviewer using a 4-point Likert scale.

Additionally, laboratory tests, clinical assessments, and functional tests were carried out. To measure comorbidity and physical frailty, a modified Functional Comorbidity Index (FCI) and the Short Physical Performance Battery (SPPB), combining tests of balance, gait speed, and 5-times chair rise, were used as described previously [27].

A loneliness score was created by asking the following question: “Sometimes one can feel lonely even if it doesn’t appear that way, because you may have many relatives. How lonely do you feel given a scale from 0 (not at all) to 10 (totally)?” More details can be found elsewhere [28].

To register each participant’s current intake of drugs, the barcodes of all drug packages in the participant’s household were scanned. Drugs without barcodes were recorded manually. All drugs were classified according to the Anatomical Therapeutic Chemical (ATC) classification system [29].

### Anticholinergic burden and drug classification

The classification of the active ingredients as having an anticholinergic effect and the assignment of an anticholinergic burden score (ABS) were based on a systematic review comparing seven expert-based rating scales for the classification of anticholinergic activity medicines [30]. The highest reported score was chosen as the final score unless a lower score was reported more than twice as often. The only exception was quetiapine, for which the mean of all reported scores was used due to inconsistent ratings. Details on the selection of the final ABS can be found in Supplement 1. Ultimately, we obtained an ABS from zero to three for each drug. This classification was applied to all drugs that were part of the regularly scheduled medication of the cohort. Drugs that were part of the on-demand medication were not considered. By this, we ensured that the calculated ABS per participant was long-term and not only a short-term effect due to sporadically taken medications, which would likely contribute less to affecting cognitive effects.

The classification of the active substances as CYP2D6 substrates or inhibitors was conducted according to the FDA, as well as the Drug Interactions Flockhart Table™ [31, 32]. An active ingredient was classified as a CYP2D6 substrate or inhibitor if it was listed in at least one of the two tables, unless this classification was solely based on in vitro studies (Supplement 1 and 2). Accordingly, substances with multiple metabolic pathways were considered as CYP2D6 substrates or inhibitors on the condition that CYP2D6 was one of the enzymes involved. Similarly, this classification was limited to the regularly scheduled medication.

### Cognition and covariates

Cognitive performance was assessed using the MMSE, with a score range from 0 to 30 [33]. Sociodemographic and clinical covariates, including age, sex, smoking status, and depression according to the Hospital Anxiety and Depression scale (HADS), were collected as part of the baseline assessments.

For each participant, a total ABS (ABS_total_) was calculated from all drugs by adding the single ABSs. Furthermore, the anticholinergic burden deriving from drugs previously classified as CYP2D6 substrates (ABS_2D6_) was calculated. By subtracting ABS_2D6_ from ABS_total_, the anticholinergic burden deriving from non-CYP2D6-metabolised drugs was evaluated (ABS_other_).

### Genotyping

Genotyping data were obtained from a previous study using the iPLEX PGx 74 and VeriDose CYP2D6 CNV Panels (both Agena Bioscience, Inc) [34]. Genotypes and phenotype groups were assigned according to Clinical Pharmacogenetics Implementation Consortium (CPIC) recommendations [35, 36]. CYP2D6 was categorised into four phenotype groups: PM, IM, NM, and UM.

### Statistical analysis

For descriptive purposes, participants were categorised as having possible cognitive impairment if their MMSE score was ≤ 24. This threshold was chosen based on German clinical guidance and German-language interpretative ranges while maintaining a descriptively interpretable subgroup size [37]. This categorisation should not be interpreted as a clinical diagnosis, as MMSE cut-offs vary depending on population and setting [38]. For transparency, descriptive comparisons using a stricter ≤ 23 MMSE cut-off are provided in Supplement 3 and 4. Continuous variables were checked for normal distribution using the Kolmogorov-Smirnov test. As almost all were not normally distributed, the median and interquartile range are presented for each variable. For categorical variables, absolute and relative frequencies are presented. Continuous variables were compared between the two groups using the Mann-Whitney U test. Categorical variables were compared using the Chi-squared test, or the Fisher-Freeman-Halton exact test, if more than 20% of the expected cell counts were 5 or less.

Quantile regression was used to examine the association between ABS_2D6_ and cognitive performance at the 25th, 50th, and 75th percentiles of the MMSE score distribution.

To account for potential confounding, we performed four models for each method in a sequential approach. Model 1 was unadjusted to estimate the crude associations. Model 2 was adjusted for ABS_other_ to differentiate the CYP2D6-specific effect from the overall anticholinergic burden. Model 3 was additionally adjusted for age (in years) and sex (female) as demographic confounders. Model 4 accounted furthermore for smoking status (current, former, never) and depression (HADS depression score), which were considered potential confounders due to their potential influence on both CYP2D6 activity and cognitive performance. The continuous variables age and HADS depression score were mean-centred for better interpretability. Models were prepared using listwise deletion, resulting in a slightly smaller study sample for model 4 (830 vs. 872 participants) due to the additional covariates.

Results of quantile regression are presented as regression coefficients with 95% confidence intervals (CIs) representing the expected change in MMSE score per one-unit increase in the respective covariate.

To test effect modification by low CYP2D6 metaboliser status (PM, IM) for the association between ABS_2D6_ and cognitive performance, interaction terms and stratified analyses were conducted. Two interaction terms with ABS_2D6_ were added in each model: one with PM status, as well as one with IM status. Because interaction analyses are often underpowered, particularly for limited subgroup sizes, an exploratory threshold of *p* < 0.2 was applied to identify potential patterns of effect modification.

Subsequently, stratified analyses were performed by dividing the study sample into three subgroups (PM, IM, NM) and running each model separately in these subgroups. The UM subgroup was excluded due to a too-small number of cases (*n* = 11) for individual analyses. For comparison, analyses were repeated by grouping low-activity phenotypes (PMs and IMs) and comparing them with non-low-activity phenotypes (NMs and UMs).

Statistical analysis was conducted in Python version 3.12.9 and R version 4.1.2.

## Results

### Study population

As depicted in Table 1, *n* = 872 participants were in the final study sample. They had a median age of 74 years, and 56.4% were male. The median MMSE score was 29, and more than 80% were in the SPPB category fit (score ≥ 10). They took a median of three drugs and had a median FCI score of two. The overall sample consisted of 43 participants (4.9%) in the group with possible cognitive impairment and 829 participants (95.1%) in the non-cognitive impairment group.

**Table 1:** Cohort description of the final study sample with available MMSE score and CYP2D6 genotypes

	Missing, *n*	Final study sample *n* = 872	Possible cognitive impairment (MMSE ≤ 24) *n* = 43 (4.9%)	Non-cognitive impairment (MMSE 30 − 25) *n* = 829 (95.1%)	*p*-value
Age in years, median (IQR)	-	74 (70; 82)	80 (72; 85)	74 (70; 81)	**0.027 **
Male, *n* (%)	-	492 (56.4)	26 (60.5)	466 (56.2)	0.696
Body mass index, median (IQR)	2	27.0 (24.9; 29.9)	27.6 (24.4; 30.8)	26.9 (24.9; 29.8)	0.569
Diabetes, n (%)	-	120 (13.8)	7 (16.3)	113 (13.6)	0.791
Hypertension, *n* (%)	1	457 (52.5)	22 (51.2)	435 (52.5)	0.985
FCI score, median (IQR)	91	2 (1; 4)	4 (3; 5)	2 (1; 4)	**< 0.001**
SPPB category, *n* (%)	72				**< 0.001**
Fit (≥ 10)		647 (80.9)	21 (55.3)	626 (82.2)	
Prefrail (7–9)		121 (15.1)	7 (18.4)	114 (15.0)	
Frail (≤ 6)		32 (4.0)	10 (26.3)	22 (2.9)	
MMSE score, median (IQR)	-	29 (27; 30)			
MMSE category, *n* (%)	-				
Normal (30 − 28)		635 (72.8)			
Mild cognitive impairment (27 − 25)		194 (22.2)			
Possible dementia (≤ 24)		43 (4.9)			
Number of drugs, median (IQR)	-	3 (2; 5)	4 (2; 7)	3 (1; 5)	0.079
Loneliness score, median (IQR)	19	0 (0; 3)	0 (0; 3)	0 (0; 3)	0.627
HADS depression score, median (IQR)	41	3 (2; 5)	6 (3; 8)	3 (2; 5)	**< 0.001**
Smoker, *n* (%)	1				0.680
Current		55 (6.3)	4 (9.3)	51 (6.2)	
Former		375 (43.1)	17 (39.5)	358 (43.2)	
Never		441 (50.6)	22 (51.2)	419 (50.6)	
Visual impairment, *n* (%)	11	131 (15.2)	13 (30.2)	118 (14.4)	**0.009**
Hearing impairment, *n* (%)	15	218 (25.4)	18 (42.9)	200 (24.5)	**0.013**
Education	7				**0.002**
≤ 9 years		495 (57.2)	34 (81.0)	461 (56.0)	
10 years		179 (20.7)	7 (16.7)	172 (20.9)	
≥ 12 years		191 (22.1)	1 (2.4)	190 (23.1)	
CYP2D6, *n* (%)	-				**0.031**
Poor metaboliser (PM)		81 (9.3)	3 (7.0)	78 (9.4)	
Intermediate metaboliser (IM)		341 (39.1)	9 (20.9)	332 (40.0)	
Normal metaboliser (NM)		439 (50.3)	31 (72.1)	408 (49.2)	
Ultrarapid metaboliser (UM)		11 (1.3)	-	11 (1.3)	

*p*-values for possible cognitive impairment/ non-cognitive impairment group comparison obtained using Mann-Whitney U tests for continuous variables and Chi-squared tests for categorical variables. Significant values in bold text

Abbreviations: *FCI* Functional Comorbidity Index, *HADS* Hospital Anxiety and Depression Scale, *IQR* interquartile range, *MMSE* Mini-Mental State Examination, *SPPB* Short Physical Performance Battery

Participants in the group with possible cognitive impairment were significantly older than those in the non-cognitive impairment group. They had significantly more comorbidities, were frailer, and had higher HADS depression scores. There were significantly higher rates of visual and hearing impairment and lower levels of education in the group with possible cognitive impairment. The number of drugs per participant was slightly higher in the group with possible cognitive impairment, although not significantly. The distribution of CYP2D6 phenotypes differed significantly between the two groups, with a higher proportion of NMs and fewer IMs in the group with possible cognitive impairment. Table 2 shows that ABS_total_ was significantly higher in the group with possible cognitive impairment, whereas ABS_2D6_ showed a non-significant trend in the same direction. Table 3 provides a detailed overview of MMSE scores and categories as well as ABS exposures across CYP2D6 metaboliser phenotypes. Notably, the median MMSE score was lowest in the PM group. The use of individual regularly scheduled anticholinergic drugs is presented in Supplement 5 for both the final study sample and stratified by cognitive status.

**Table 2:** Characteristics of anticholinergic drug use in the final study sample and stratified by cognitive status

	Missing, *n*	Final study sample *n* = 872	Possible cognitive impairment (MMSE ≤ 24) *n* = 43 (4.9%)	Non-cognitive impairment (MMSE 30 − 25) *n* = 829 (95.1%)	*p*-value
Total anticholinergic burden score (ABS_total_), median (IQR)	-	0 (0; 1)	1 (0; 2)	0 (0; 1)	**0.011**
Anticholinergic burden score of drugs metabolised by CYP2D6 (ABS_2D6_), *n* (%)	-				0.060
0		725 (83.1)	33 (76.7)	692 (83.5)	
1		119 (13.6)	6 (14.0)	113 (13.6)	
2		7 (0.8)	2 (4.7)	5 (0.6)	
3		16 (1.8)	2 (4.7)	14 (1.7)	
4–5		5 (0.6)	-	5 (0.6)	
Anticholinergic CYP2D6 substrates, *n* (%)	-				0.414
0		725 (83.1)	33 (76.7)	692 (83.5)	
1		141 (16.2)	10 (23.3)	131 (15.8)	
2–3		6 (0.7)	-	6 (0.7)	

*p*-values for possible cognitive impairment/ non-cognitive impairment group comparison obtained using Mann-Whitney U tests for continuous variables and Fisher-Freeman-Halton Exact tests for categorical variables. Significant values in bold text

Abbreviations: *MMSE* Mini-Mental State Examination

**Table 3:** Characteristics of CYP2D6 metaboliser phenotype groups in the study cohort (*n* = 872)

	CYP2D6 poor metaboliser (*n* = 81, 9.3%)	CYP2D6 intermediate metaboliser (*n* = 341, 39.1%)	CYP2D6 normal metaboliser (*n* = 439, 50.3%)	CYP2D6 ultrarapid metaboliser (*n* = 11, 1.3%)
MMSE score, median (IQR)	28 (27; 29)	29 (27; 30)	29 (27; 29)	29 (27; 29.5)
MMSE category, n (%)				
Non-cognitive impairment (30 − 25)	78 (96.3)	332 (97.4)	408 (92.9)	11 (100)
Possible cognitive impairment (≤ 24)	3 (3.7)	9 (2.6)	31 (7.1)	0 (0)
Participants with ABS_2D6_ > 0, n	15	57	73	2

Abbreviations: *ABS*_*2D6*_ anticholinergic burden score deriving from drugs metabolised by CYP2D6, *IQR* interquartile range, *MMSE* Mini-Mental State Examination

### Multiple quantile regression analysis

For better readability, Table 4 presents only the fully adjusted quantile regression models; the complete results, including all sequential models, are provided in Supplement 6, 7, and 8. Quantile regression analyses for models 1 and 2 showed a significant association between ABS_2D6_ and lower MMSE scores at the 50th and 75th percentiles of the MMSE score distribution (coefficients for model 2: β_0.50_ = -0.33 [95% CI: -0.56, -0.11], β_0.75_ = -0.33 [95% CI: -0.51, -0.16]; Supplement 6). This association was not significant in models 3 and 4 (Table 4).

**Table 4:** Fully adjusted quantile regression models (model 4) for the association of anticholinergic burden deriving from drugs metabolised by CYP2D6 with MMSE score in the overall sample and stratified by CYP2D6 metaboliser status (primary analysis: *n* = 830; secondary analysis: *n* = 816)

Analysis	Sample	25th percentile of MMSE score, ABS_2D6_ β (95% CI)	50th percentile of MMSE score, ABS_2D6_ β (95% CI)	75th percentile of MMSE score, ABS_2D6_ β (95% CI)
Primary analysis	Overall sample	-0.00 (-0.28 to 0.28)	-0.16 (-0.39 to 0.06)	-0.04 (-0.21 to 0.14)
	CYP2D6 PM	-0.05 (-0.63 to 0.53)	-0.32 (-0.98 to 0.34)	-0.03 (-0.60 to 0.54)
	CYP2D6 IM	0.03 (-0.44 to 0.50)	-0.35 (-0.75 to 0.05)	-0.34* **(-0.64 to -0.05)**
	CYP2D6 NM	-0.02 (-0.41 to 0.36)	0.04 (-0.24 to 0.33)	0.05 (-0.22 to 0.31)
Secondary analysis	Overall smaple	-0.01 (-0.32 to 0.31)	-0.08 (-0.33 to 0.17)	0.00 (-0.19 to 0.19)
	CYP2D6 PM	-0.04 (-0.76 to 0.68)	-0.61 (-1.35 to 0.12)	-0.10 (-0.57 to 0.38)
	CYP2D6 IM	0.03 (-0.49 to 0.55)	-0.36 (-0.80 to 0.09)	-0.50* **(-0.82 to -0.18)**
	CYP2D6 NM	0.04 (-0.39 to 0.46)	0.18 (-0.13 to 0.49)	0.05 (-0.21 to 0.30)

Significant values in bold text. *p < 0.05, **p < 0.01, ***p < 0.001

Abbreviations: *ABS*_*2D6*_ anticholinergic burden score deriving from drugs metabolised by CYP2D6, *CI* confidence interval, *IM* intermediate metaboliser, *MMSE* Mini-Mental State Examination, *NM* normal metaboliser, *PM* poor metaboliser

All coefficients represent the expected change in MMSE score per one-unit increase in ABS_2D6_. The secondary analysis excluded participants with concurrent use of CYP2D6 inhibitors and CYP2D6-metabolised anticholinergic drugs

A significant effect modification by CYP2D6 metaboliser status was consistently observed for IM status at the 75th percentile, with a p-value of 0.012 in model 4. In the stratified analysis, IMs consistently showed a significant negative association between ABS_2D6_ and MMSE score at the 75th percentile, with a coefficient of -0.34 [95% CI: -0.64, -0.05] in the fully adjusted model. An additional significant interaction term for IM status was observed at the 50th percentile in model 4 (*p* = 0.052). While the corresponding coefficient in stratified analyses was not significant, IMs suggested a negative association between ABS_2D6_ and MMSE score at the 50th percentile across all models, with a coefficient of -0.35 [95% CI: -0.75, 0.05] in model 4. There was no significant effect modification by PM or NM status, and no significant association between ABS_2D6_ and MMSE score in both groups in stratified analyses. When grouping low-activity (PMs and IMs) versus non-low-activity phenotypes (NMs and UMs), significant interaction terms for low-activity phenotypes were observed consistently at the 75th percentile (model 4: *p* = 0.012) and for models 3 and 4 at the 50th percentile (model 4: *p* = 0.038). In stratified analyses using this pooled grouping, low-activity phenotypes showed a consistent negative association between ABS_2D6_ and MMSE score at the 75th percentile across all models (model 4: β = -0.47 [95% CI: -0.72, -0.21]; Supplement 8).

In the fully adjusted quantile regression model, ABS_other_ was significantly associated with lower MMSE scores at all examined percentiles (coefficients: β_0.25_ = -0.25 [95% CI: -0.40, -0.10], β_0.50_ = -0.17 [95% CI: -0.28, -0.05], β_0.75_ = -0.17 [95% CI: -0.25, -0.10]; Table 5). Age was also significantly associated with lower MMSE scores at all examined percentiles (coefficients: β_0.25_ = -0.06, β_0.50_ = -0.04, β_0.75_ = -0.05). Sex and smoking status showed no significant association with the MMSE score. The HADS depression score was only significantly associated with lower MMSE scores at the 25th percentile (β = -0.08 [95% CI: -0.15, -0.02]).

**Table 5:** Fully adjusted quantile regression model (model 4; *n* = 830): predictors of MMSE score

Predictor	25th percentile of MMSE score, β (95% CI)	50th percentile of MMSE score, β (95% CI)	75th percentile of MMSE score, β (95% CI)
ABS_2D6_	-0.00 (-0.28 to 0.28)	-0.16 (-0.39 to 0.06)	-0.04 (-0.21 to 0.14)
ABS_other_	-0.25** **(-0.40 to -0.10)**	-0.17** **(-0.28 to -0.05)**	-0.17*** **(-0.25 to -0.10)**
Age (centred)	-0.06*** **(-0.08 to -0.03)**	-0.04*** **(-0.07 to -0.02)**	-0.05*** **(-0.07 to -0.03)**
Sex (female)	0.20 (-0.17 to 0.56)	-0.03 (-0.33 to 0.27)	0.03 (-0.19 to 0.24)
Smoking status	-0.05 (-0.36 to 0.25)	-0.08 (-0.32 to 0.16)	-0.04 (-0.20 to 0.13)
HADS depression score (ascending, centred)	-0.08** **(-0.15 to -0.02)**	-0.02 (-0.07 to 0.02)	0.01 (-0.03 to 0.04)

Significant values in bold text. *p < 0.05, **p < 0.01, ***p < 0.001

Abbreviations: *ABS*_*2D6*_ anticholinergic burden score deriving from drugs metabolised by CYP2D6, *ABS*_*other*_ anticholinergic burden score deriving from non-CYP2D6-metabolised drugs, *HADS* Hospital Anxiety and Depression Scale, *MMSE* Mini-Mental State Examination

All continuous predictors were mean-centred

### Secondary analysis accounting for potential phenoconversion

In the secondary analysis, participants with concurrent use of at least one CYP2D6 inhibitor and a CYP2D6-metabolised anticholinergic drug (i.e., ABS_2D6_ > 0) were excluded to account for potential phenoconversion, meaning a shift towards lower CYP2D6 activity due to drug-drug interactions, leaving 857 participants in models 1–3 and 816 in model 4. The associations between ABS_2D6_ and lower MMSE scores were comparable to those of the primary analysis. Additionally, a significant association with lower MMSE scores was observed at the 25th percentile in model 2 (β = -0.25 [95% CI: -0.50, -0.00], Supplement 7).

Notably, the effect modification by CYP2D6 metaboliser status became more pronounced: for IMs, the interaction term at the 75th percentile of the MMSE score distribution remained significant across all models, with *p* = 0.022 in the fully adjusted model. In stratified analyses, the negative association between ABS_2D6_ and MMSE score at the 75th percentile remained significant for IMs across all models, with a stronger coefficient of -0.50 [95% CI: -0.82, -0.18] compared to the primary analysis in the fully adjusted model. Additionally, the interaction term for IM status at the 50th percentile was now significant across all models (model 4: *p* = 0.022), and stratified analyses showed significant coefficients in models 1 and 2 (both − 0.50).

For PMs, significant interaction terms emerged at the 50th percentile in models 2 and 4 (*p* = 0.120 and *p* = 0.128) and at the 75th percentile in models 1 and 2 (*p* < 0.001 and *p* = 0.071). The corresponding coefficients in stratified analyses were stronger than in the overall sample, but only reached significance for model 1 at the 75th percentile (β = -0.75 [95% CI: -1.33, -0.17]).

## Discussion

In this cross-sectional analysis, anticholinergic burden deriving from regularly taken drugs was associated with poorer cognitive performance, captured by MMSE scores. The initially significant association between the anticholinergic burden deriving from drugs metabolised by CYP2D6 (ABS_2D6_) and poorer cognition at the 50th and 75th percentiles of the MMSE score distribution did not persist after adjustment for age and sex. This may reflect the stronger impact of these factors on cognitive performance. There was a consistently significant effect modification for IM status at the 75th percentile of the MMSE score distribution. This finding should be interpreted as exploratory, given the limited subgroup sizes and the restricted variability of MMSE scores in this relatively healthy cohort. At the 50th percentile, the coefficients among IMs pointed in a similar negative direction but did not persistently reach statistical significance, whereas coefficients at the 25th percentile were less consistent. The overall inconsistency outside of the 75th percentile may be attributable to the ceiling effect of the MMSE score and the stronger influence of other covariates (i.e., ABS_other_ and HADS depression score) in the lower range of the score distribution. For PMs, coefficients were generally negative but imprecise and not consistently statistically significant, which likely reflects limited statistical power due to the substantially smaller sample size in this group. This increases the risk of false-negative findings, and although stronger effects in PMs would be pharmacologically plausible, the present dataset does not allow for assessing this subgroup reliably. Overall, the observed effect modification among IMs provides tentative support for our hypothesis that CYP2D6 metaboliser status may modify the association between ABS_2D6_ and cognitive performance.

Our results are in line with previous studies reporting a general association between anticholinergic burden and poorer cognitive performance in older adults [10–14]. Our results complement findings from previous research reporting higher serum anticholinergic activity (SAA) in older CYP2D6 PMs with high anticholinergic burden [25]. Although our study did not measure SAA levels, this previous finding provides a plausible pharmacological context for investigating CYP2D6 activity in relation to anticholinergic burden and cognition.

While there are several studies analysing the association of anticholinergic load of drugs on cognitive function, those studies usually do not respect individual risk factors of patients [39]. This leads to general recommendations to avoid anticholinergic drugs in older adults [40]. However, sometimes a drug treatment with an anticholinergic drug inevitably needs to be administered. Hence, it is important to assess the individual risk more precisely. While the summative effect of drugs is a well-known concept, we now introduce individual genetically predicted variability as a risk factor. As our study focuses on genetic variability in drug-metabolising capacity, it is important to mention that drugs can also decrease the CYP2D6 activity. The use of other substrates or CYP2D6-inhibiting drugs can therefore cause a CYP2D6 NM to become phenotypically an IM or PM. Such phenoconversion may lead to discrepancies between genotype-predicted and actual metaboliser status [41]. A secondary analysis excluding participants with potential phenoconversion showed a more pronounced effect modification by CYP2D6 metaboliser status among IMs, further supporting a potential modifying role of CYP2D6 metabolic variability. In PMs, the estimates generally pointed in the expected direction but remained uncertain due to the small subgroup size and should be interpreted with caution.

There are limitations that need to be considered. First, statistical power was limited due to the fragmented composition of the study sample. Particularly in stratified analyses, the limited number of participants with ABS_2D6_ greater than zero was further divided by CYP2D6 phenotype, resulting in small subgroups. This problem is especially evident in PMs, likely increasing the risk of false-negative findings and unstable effect estimates. Therefore, the subgroup and interaction analyses should be interpreted as exploratory and hypothesis-generating.

Second, the study sample consisted of relatively healthy, community-dwelling older adults due to the needed ability for informed consent for participation in the study. The resulting high MMSE scores and restricted cognitive variability may have limited the ability to detect clinically meaningful associations.

Third, the cognitive outcome was measured using the MMSE, which is a global cognitive screening instrument. This suggests that subtle cognitive effects associated with anticholinergic burden may have remained undetected, potentially leading to an underestimation of associations. Cognitive domains such as executive function and working memory may be particularly relevant in the context of anticholinergic burden and should be assessed in future studies.

Fourth, due to the cross-sectional study design, temporal relationships and causal conclusions are not possible. In addition, residual confounding and indication bias cannot be excluded. Fifth, the baseline data were collected between 2009 and 2010. While the fundamental pharmacogenetic question remains relevant, prescribing patterns and awareness of anticholinergic burden likely changed, which may limit the current applicability of the findings. Furthermore, there are several methods for classifying drugs as CYP2D6 substrates and assigning an ABS, which may lead to varying results [42]. However, recent findings suggest that broader-coverage anticholinergic burden scales offer a more comprehensive risk assessment, although no single scale clearly outperforms others in predicting cognitive outcomes [43]. Finally, phenotype assignment based on genotype is inherently limited, particularly for the highly polymorphic CYP2D6 gene, possibly resulting in errors due to hybrid or rare alleles [44].

Notwithstanding these limitations, our study is among the first to examine the association between anticholinergic burden and cognitive impairment using a pharmacogenetic approach. The observed effect modification among IMs at the 75th percentile of the MMSE score distribution provides a first indication that CYP2D6 metaboliser status may be relevant in this context. Accounting for phenoconversion is a clear strength of our study [24, 45]. In addition, the population-based design, including community-dwelling older adults, provides insight into a relatively healthy older population, while generalisability to frailer and more cognitively impaired populations remains limited.

In conclusion, our findings provide exploratory evidence that CYP2D6 metaboliser status may be relevant for interindividual susceptibility to anticholinergic burden-related lower cognitive performance. These results require confirmation in larger studies with higher levels of anticholinergic exposure, broader cognitive ranges, and preferably longitudinal designs including more sensitive cognitive assessments.

## Supplementary Information

Below is the link to the electronic supplementary material.

Supplementary Material 1 (DOCX 50.8 KB)

## Data Availability

The datasets analysed during the current study are not publicly available due to the conditions provided in the informed consent at the time of recruitment but are available from the corresponding author on reasonable request.
