# Supplementary material for: Cognitive function in the context of pharmacogenetic CYP2D6 variability and anticholinergic burden in older adults – results from the ActiFE study
Source: Eur J Clin Pharmacol. 2026 Aug 4;82(8):225. doi: 10.1007/s00228-026-04155-y (PMC13433654; doi:10.1007/s00228-026-04155-y)
Supplement: Supplementary file 1 [file 228_2026_4155_MOESM1_ESM.docx]

**Supplementary Information**

**Title:** Cognitive function in the context of pharmacogenetic CYP2D6 variability and anticholinergic burden in older adults – results from the ActiFE study

**Short title:** CYP2D6 variability on anticholinergics

**Journal:** European Journal of Clinical Pharmacology

**Authors:** Linda Lorenz, Judith Berres, Michael D Denkinger, Dietrich Rothenbacher, Stefanie Braig, Dhayana Dallmeier, Katja S Just

**Corresponding author:** Katja Susanne Just - Institute of Clinical Pharmacology, University Hospital RWTH Aachen, Aachen, Germany (kjust@ukaachen.de)

**Supplement 1:** List of anticholinergic drugs, final anticholinergic burden scores, and CYP2D6 substrate classification used in this study

| Drug | Reported score by Salahudeen et al. [1] (no. of studies supporting score) | Final Score | CYP2D6 substrate according to FDA and/or Flockhart [2, 3] |
| --- | --- | --- | --- |
| Amantadine | 2 (2), 1 (2) | 2 |  |
| Amitriptyline | 3 | 3 | x |
| Atenolol | 1 | 1 |  |
| Azathioprine | 1 | 1 |  |
| Baclofen | 2 | 2 |  |
| Benazepril | 1 | 1 |  |
| Betaxolol | 1 | 1 |  |
| Bisacodyl | 1 | 1 |  |
| Butylscopolamine | 3 | 3 |  |
| Captopril | 1 | 1 |  |
| Carbamazepine | 2 (2), 1 (1) | 2 |  |
| Carbidopa | 1 | 1 |  |
| Celecoxib | 1 | 1 |  |
| Cetirizine | 2 (3), 1 (1) | 2 |  |
| Chlortalidone | 1 | 1 |  |
| Citalopram | 1 | 1 |  |
| Clomipramine | 3 | 3 | x |
| Clonazepam | 1 | 1 |  |
| Codeine | 2 (1), 1 (4) | 1 | x |
| Colchicine | 3 (1), 1 (1) | 3 |  |
| Darifenacin | 3 | 3 |  |
| Dexamethasone | 1 | 1 |  |
| Digitoxin | 1 | 1 |  |
| Digoxin | 3 (1), 1 (4) | 1 |  |
| Diltiazem | 1 | 1 |  |
| Dimenhydrinate | 3 | 3 |  |
| Dipyridamole | 1 | 1 |  |
| Doxepin | 3 | 3 | x |
| Entacapone | 1 | 1 |  |
| Escitalopram | 1 | 1 | x |
| Fentanyl | 1 | 1 |  |
| Fesoterodine | 3 | 3 |  |
| Fexofenadine | 2 | 2 |  |
| Flurazepam | 1 | 1 |  |
| Fluticasone-salmeterol | 1 | 1 |  |
| Furosemide | 3 (1), 1 (2) | 3 |  |
| Hydrocortisone | 1 | 1 |  |
| Hydroxyzine | 3 | 3 |  |
| Ipratropium (including inhalative) | 3 | 3 |  |
| Isosorbide (including mono-/dinitrate) | 1 | 1 |  |
| Levocetirizine | 1 | 1 |  |
| Levomepromazine | 3 | 3 |  |
| Lithium | 1 | 1 |  |
| Loperamide | 2 (1), 1 (4) | 1 |  |
| Lorazepam | 1 | 1 |  |
| Metformin | 1 | 1 |  |
| Methotrexate | 1 | 1 |  |
| Methylprednisolone | 1 | 1 |  |
| Metoclopramide | 1 | 1 | x |
| Metoprolol | 1 | 1 | x |
| Mirtazapine | 1 | 1 |  |
| Nifedipine | 1 | 1 |  |
| Opipramol | 3 | 3 |  |
| Oxazepam | 1 | 1 |  |
| Oxcarbazepine | 2 | 2 |  |
| Oxybutynin | 3 (5), 2 (1) | 3 |  |
| Oxycodone | 1 | 1 | x |
| Paroxetine | 3 (1), 2 (3), 1 (2) | 2 | x |
| Pramipexole | 1 | 1 |  |
| Prednisolone | 1 | 1 |  |
| Prednisone | 1 | 1 |  |
| Promethazine | 3 | 3 | x |
| Propiverine | 3 | 3 |  |
| Pseudoephedrine | 2 | 2 |  |
| Quetiapine | 3 (1), 2 (1), 1 (2) | 2 |  |
| Ranitidine | 2 (2), 1 (4) | 2 |  |
| Risperidone | 1 | 1 | x |
| Selegiline | 1 | 1 |  |
| Sertraline | 1 | 1 |  |
| Solifenacin | 3 | 3 |  |
| Sumatriptan | 1 | 1 |  |
| Theophylline | 2 (2), 1 (3) | 2 |  |
| Tizanidine | 3 | 3 |  |
| Tolterodine | 3 (5), 2 (1) | 3 | x |
| Tramadol | 2 (2), 1 (1) | 2 | x |
| Trandolapril | 1 | 1 |  |
| Triamcinolone | 1 | 1 |  |
| Triamterene | 1 | 1 |  |
| Trimipramine | 3 | 3 | x |
| Trospium | 3 | 3 |  |
| Valproic acid | 1 | 1 |  |
| Venlafaxine | 1 | 1 | x |
| Warfarin | 1 | 1 |  |

83 of the drugs used at baseline in the ActiFE study were found in the 195 drugs listed by Salahudeen et al. and therefore assigned a final anticholinergic burden score.

For some drugs, more than one anticholinergic burden score was given. The highest reported score was always chosen as the final score, unless a lower score was reported more than twice as often. The only exception to this rule is quetiapine, for which the mean of all reported scores was chosen as the final score.

15 of the anticholinergic drugs were classified as CYP2D6 substrates.

**Supplement 2:** CYP2D6 inhibitors used at baseline in this study according to the FDA and the Drug Interactions Flockhart Table^TM^ [2, 3]:

Amiodarone, Celecoxib, Citalopram, Clomipramine, Doxepin, Duloxetine, Escitalopram, Hydroxychloroquine, Levomepromazine, Paroxetine, Sertraline, Terbinafine.

**Supplement 3:** Cohort description of the final study sample with available MMSE score and CYP2D6 genotypes using an alternative lower MMSE cut-off

|  | Missing, n | Final study sample n = 872 | Possible cognitive impairment  (MMSE ≤ 23)  n = 20 (2.3%) | Non-cognitive impairment  (MMSE 30-24) n = 852 (97.7%) | p-value |
| --- | --- | --- | --- | --- | --- |
| Age in years, median (IQR) | - | 74 (70; 82) | 78 (71; 82) | 74 (70; 82) | 0.328 |
| Male, n (%) | - | 492 (56.4) | 11 (55.0) | 481 (56.5) | 1.000 |
| Body mass index, median (IQR) | 2 | 27.0 (24.9; 29.9) | 28.5 (24.6; 31.1) | 27.0 (24.9; 29.8) | 0.417 |
| Diabetes, n (%) | - | 120 (13.8) | 4 (20.0) | 116 (13.6) | 0.505 |
| Hypertension, n (%) | 1 | 457 (52.5) | 9 (45.0) | 448 (52.6) | 0.653 |
| FCI score, median (IQR) | 91 | 2 (1; 4) | 5 (3; 5) | 2 (1; 4) | **<0.001** |
| SPPB category, n (%) | 72 |  |  |  | **<0.001** |
| Fit (≥ 10) |  | 647 (80.9) | 9 (45.0) | 638 (81.8) |  |
| Prefrail (7-9) |  | 121 (15.1) | 5 (25.0) | 116 (14.9) |  |
| Frail (≤ 6) |  | 32 (4.0) | 6 (30.0) | 26 (3.3) |  |
| MMSE score, median (IQR) | - | 29 (27; 30) |  |  |  |
| Number of drugs, median (IQR) | - | 3 (2; 5) | 4 (3; 7) | 3 (1; 5) | **0.029** |
| Loneliness score, median (IQR) | 19 | 0 (0; 3) | 2 (0; 4) | 0 (0; 3) | 0.185 |
| HADS depression score, median (IQR) | 41 | 3 (2; 5) | 8 (6; 8) | 3 (2; 5) | **<0.001** |
| Smoker, n (%) | 1 |  |  |  | 0.252 |
| Current |  | 55 (6.3) | 3 (15.0) | 52 (6.1) |  |
| Former |  | 375 (43.1) | 7 (35.0) | 368 (43.2) |  |
| Never |  | 441 (50.6) | 10 (50.0) | 431 (50.6) |  |
| Visual impairment, n (%) | 11 | 131 (15.2) | 6 (30.0) | 125 (14.9) | 0.104 |
| Hearing impairment, n (%) | 15 | 218 (25.4) | 9 (45.0) | 209 (25.0) | 0.076 |
| Education | 7 |  |  |  | 0.078 |
| ≤ 9 years |  | 495 (57.2) | 16 (80.0) | 479 (56.7) |  |
| 10 years |  | 179 (20.7) | 3 (15.0) | 176 (20.8) |  |
| ≥ 12 years |  | 191 (22.1) | 1 (5.0) | 190 (22.5) |  |
| CYP2D6, n (%) | - |  |  |  | 0.437 |
| Poor metaboliser (PM) |  | 81 (9.3) | 1 (5.0) | 80 (9.4) |  |
| Intermediate metaboliser (IM) |  | 341 (39.1) | 5 (25.0) | 336 (39.4) |  |
| Normal metaboliser (NM) |  | 439 (50.3) | 14 (70.0) | 425 (49.9) |  |
| Ultrarapid metaboliser (UM) |  | 11 (1.3) | - | 11 (1.3) |  |

p-values for possible cognitive impairment/ non-cognitive impairment group comparison obtained using Mann-Whitney U tests for continuous variables and Chi-squared tests or Fisher’s Exact/ Fisher-Freeman-Halton test for categorical variables, as appropriate depending on expected cell counts. Significant values in bold text.

Abbreviations: FCI: Functional Comorbidity Index; HADS: Hospital Anxiety and Depression Scale; IQR: interquartile range; MMSE: Mini-Mental State Examination; SPPB: Short Physical Performance Battery

**Supplement 4:** Characteristics of anticholinergic drug use in the final study sample and stratified by cognitive status using an alternative lower MMSE cut-off

|  | Missing, n | Final study sample n = 872 | Possible cognitive impairment  (MMSE ≤ 23)  n = 20 (2.3%) | Non-cognitive impairment  (MMSE 30-24) n = 852 (97.7%) | p-value |
| --- | --- | --- | --- | --- | --- |
| Total anticholinergic burden score (ABS_total_), median (IQR) | - | 0 (0; 1) | 0 (0; 3) | 0 (0; 1) | **0.043** |
| Anticholinergic burden score of drugs metabolised by CYP2D6 (ABS_2D6_), n (%) | - |  |  |  | **0.003** |
| 0 |  | 725 (83.1) | 15 (75.0) | 710 (83.3) |  |
| 1 |  | 119 (13.6) | 1 (5.0) | 118 (13.8) |  |
| 2 |  | 7 (0.8) | 2 (10.0) | 5 (0.6) |  |
| 3 |  | 16 (1.8) | 2 (10.0) | 14 (1.6) |  |
| 4-5 |  | 5 (0.6) | - | 5 (0.6) |  |
| Anticholinergic CYP2D6 substrates, n (%) | - |  |  |  | 0.436 |
| 0 |  | 725 (83.1) | 15 (75.0) | 710 (83.3) |  |
| 1 |  | 141 (16.2) | 5 (25.0) | 136 (16.0) |  |
| 2-3 |  | 6 (0.7) | - | 6 (0.7) |  |

p-values for possible cognitive impairment/ non-cognitive impairment group comparison obtained using Mann-Whitney U tests for continuous variables and Fisher-Freeman-Halton Exact tests for categorical variables. Significant values in bold text.

Abbreviations: MMSE: Mini-Mental State Examination

**Supplement 5:** Regularly scheduled anticholinergic drug use in the final study sample (n = 872) and stratified by cognitive status

|  | Final study sample n = 872 | Possible cognitive impairment  (MMSE ≤ 24)  n = 43 (4.9%) | Non-cognitive impairment  (MMSE 30-25) n = 829 (95.1%) |
| --- | --- | --- | --- |
| **No CYP2D6 substrate** |  |  |  |
| Amantadine, n (%) | 4 (0.5) | 1 (2.3) | 3 (0.4) |
| Atenolol, n (%) | 8 (0.9) | 0 (0.0) | 8 (1.0) |
| Azathioprine, n (%) | 4 (0.5) | 0 (0.0) | 4 (0.5) |
| Baclofen, n (%) | 1 (0.1) | 0 (0.0) | 1 (0.1) |
| Benazepril, n (%) | 2 (0.2) | 0 (0.0) | 2 (0.2) |
| Betaxolol, n (%) | 1 (0.1) | 0 (0.0) | 1 (0.1) |
| Captopril, n (%) | 11 (1.3) | 1 (2.3) | 10 (1.2) |
| Carbamazepine, n (%) | 6 (0.7) | 1 (2.3) | 5 (0.6) |
| Carbidopa, n (%) | 2 (0.2) | 1 (2.3) | 1 (0.1) |
| Celecoxib, n (%) | 1 (0.1) | 0 (0.0) | 1 (0.1) |
| Cetirizine, n (%) | 2 (0.2) | 0 (0.0) | 2 (0.2) |
| Chlortalidone, n (%) | 1 (0.1) | 0 (0.0) | 1 (0.1) |
| Citalopram, n (%) | 5 (0.6) | 0 (0.0) | 5 (0.6) |
| Clonazepam, n (%) | 1 (0.1) | 0 (0.0) | 1 (0.1) |
| Darifenacin, n (%) | 1 (0.1) | 1 (2.3) | 0 (0.0) |
| Dexamethasone, n (%) | 1 (0.1) | 0 (0.0) | 1 (0.1) |
| Digitoxin, n (%) | 8 (0.9) | 1 (2.3) | 7 (0.8) |
| Digoxin, n (%) | 1 (0.1) | 0 (0.0) | 1 (0.1) |
| Diltiazem, n (%) | 3 (0.3) | 0 (0.0) | 3 (0.4) |
| Dimenhydrinate, n (%) | 1 (0.1) | 1 (2.3) | 0 (0.0) |
| Dipyridamole, n (%) | 5 (0.6) | 0 (0.0) | 5 (0.6) |
| Entacapone, n (%) | 2 (0.2) | 0 (0.0) | 2 (0.2) |
| Fentanyl, n (%) | 2 (0.2) | 0 (0.0) | 2 (0.2) |
| Fluticasone-salmeterol, n (%) | 16 (1.8) | 1 (2.3) | 15 (1.8) |
| Furosemide, n (%) | 30 (3.4) | 3 (7.0) | 27 (3.3) |
| Hydrocortisone, n (%) | 2 (0.2) | 0 (0.0) | 2 (0.2) |
| Ipratropium, n (%) | 7 (0.8) | 1 (2.3) | 6 (0.7) |
| Isosorbide, n (%) | 9 (1.0) | 0 (0.0) | 9 (1.1) |
| Levomepromazine, n (%) | 1 (0.1) | 0 (0.0) | 1 (0.1) |
| Lithium, n (%) | 3 (0.3) | 1 (2.3) | 2 (0.2) |
| Lorazepam, n (%) | 1 (0.1) | 0 (0.0) | 1 (0.1) |
| Metformin, n (%) | 58 (6.7) | 3 (7.0) | 55 (6.6) |
| Methotrexate, n (%) | 4 (0.5) | 0 (0.0) | 4 (0.5) |
| Mirtazapine, n (%) | 3 (0.3) | 0 (0.0) | 3 (0.4) |
| Nifedipine, n (%) | 7 (0.8) | 0 (0.0) | 7 (0.8) |
| Opipramol, n (%) | 3 (0.3) | 0 (0.0) | 3 (0.4) |
| Oxazepam, n (%) | 1 (0.1) | 0 (0.0) | 1 (0.1) |
| Oxcarbazepine, n (%) | 2 (0.2) | 0 (0.0) | 2 (0.2) |
| Oxybutynin, n (%) | 3 (0.3) | 0 (0.0) | 3 (0.4) |
| Pramipexole, n (%) | 6 (0.7) | 1 (2.3) | 5 (0.6) |
| Prednisolone, n (%) | 17 (1.9) | 2 (4.7) | 15 (1.8) |
| Prednisone, n (%) | 2 (0.2) | 0 (0.0) | 2 (0.2) |
| Propiverine, n (%) | 2 (0.2) | 0 (0.0) | 2 (0.2) |
| Pseudoephedrine, n (%) | 1 (0.1) | 0 (0.0) | 1 (0.1) |
| Quetiapine, n (%) | 2 (0.2) | 1 (2.3) | 1 (0.1) |
| Ranitidine, n (%) | 4 (0.5) | 0 (0.0) | 4 (0.5) |
| Selegiline, n (%) | 1 (0.1) | 0 (0.0) | 1 (0.1) |
| Sertraline, n (%) | 5 (0.6) | 0 (0.0) | 5 (0.6) |
| Solifenacin, n (%) | 2 (0.2) | 0 (0.0) | 2 (0.2) |
| Theophylline, n (%) | 7 (0.8) | 1 (2.3) | 6 (0.7) |
| Tizanidine, n (%) | 1 (0.1) | 0 (0.0) | 1 (0.1) |
| Triamterene, n (%) | 19 (2.2) | 3 (7.0) | 16 (1.9) |
| Trospium, n (%) | 3 (0.3) | 0 (0.0) | 3 (0.4) |
| Valproic acid, n (%) | 2 (0.2) | 0 (0.0) | 2 (0.2) |
| Warfarin, n (%) | 3 (0.3) | 0 (0.0) | 3 (0.4) |
|  |  |  |  |
| **CYP2D6 substrate** |  |  |  |
| Amitriptyline, n (%) | 8 (0.9) | 1 (2.3) | 7 (0.8) |
| Clomipramine, n (%) | 1 (0.1) | 0 (0.0) | 1 (0.1) |
| Doxepin, n (%) | 5 (0.6) | 1 (2.3) | 4 (0.5) |
| Escitalopram, n (%) | 3 (0.3) | 0 (0.0) | 3 (0.4) |
| Metoclopramide, n (%) | 3 (0.3) | 0 (0.0) | 3 (0.4) |
| Metoprolol, n (%) | 115 (13.2) | 6 (14.0) | 109 (13.1) |
| Oxycodone, n (%) | 1 (0.1) | 0 (0.0) | 1 (0.1) |
| Paroxetine, n (%) | 2 (0.2) | 1 (2.3) | 1 (0.1) |
| Promethazine, n (%) | 1 (0.1) | 0 (0.0) | 1 (0.1) |
| Tramadol, n (%) | 4 (0.5) | 1 (2.3) | 3 (0.4) |
| Trimipramine, n (%) | 6 (0.7) | 0 (0.0) | 6 (0.7) |
| Venlafaxine, n (%) | 6 (0.7) | 0 (0.0) | 6 (0.7) |

Abbreviations: MMSE: Mini-Mental State Examination

**Supplement 6:** Quantile regression models for the association of anticholinergic burden deriving from drugs metabolised by CYP2D6 with MMSE score

|  | Model 1,  β (95% CI),  n = 872 | Model 2,  β (95% CI),  n = 872 | Model 3,  β (95% CI),  n = 872 | Model 4,  β (95% CI),  n = 830 |
| --- | --- | --- | --- | --- |
| *25^th^ percentile of MMSE score* | | | | |
| ABS_2D6_ | -0.00  (-0.39 to 0.39) | -0.06  (-0.42 to 0.30) | -0.05  (-0.32 to 0.21) | -0.00  (-0.28 to 0.28) |
| CYP2D6  PM | -0.00  (-0.79 to 0.79) | -0.21  (-1.06 to 0.63) | -0.19  (-1.05 to 0.67) | -0.05  (-0.63 to 0.53) |
| CYP2D6  IM | -0.00  (-0.67 to 0.67) | -0.00  (-0.50 to 0.50) | -0.25  (-0.70 to 0.20) | 0.03  (-0.44 to 0.50) |
| CYP2D6  NM | -0.00  (-0.65 to 0.65) | -0.03  (-0.31 to 0.25) | 0.00  (-0.35 to 0.35) | -0.02  (-0.41 to 0.36) |
| *50^th^ percentile of MMSE score* | | | | |
| ABS_2D6_ | **-0.33****  **(-0.58 to -0.08)** | **-0.33****  **(-0.56 to -0.11)** | -0.21  (-0.43 to 0.02) | -0.16  (-0.39 to 0.06) |
| CYP2D6  PM | -0.33  (-0.98 to 0.31) | -0.33  (-0.99 to 0.33) | -0.33  (-1.01 to 0.35) | -0.32  (-0.98 to 0.34) |
| CYP2D6  IM | **-0.50****  **(-0.87 to -0.13)** | -0.33  (-0.72 to 0.05) | -0.23  (-0.62 to 0.15) | -0.35  (-0.75 to 0.05) |
| CYP2D6  NM | -0.33  (-0.67 to 0.00) | -0.08  (-0.35 to 0.18) | -0.01  (-0.30 to 0.27) | 0.04  (-0.24 to 0.33) |
| *75^th^ percentile of MMSE score* | | | | |
| ABS_2D6_ | **-0.50*****  **(-0.71 to -0.29)** | **-0.33*****  **(-0.51 to -0.16)** | -0.08  (-0.24 to 0.09) | -0.04  (-0.21 to 0.14) |
| CYP2D6  PM | -0.00  (-0.48 to 0.48) | -0.33  (-0.88 to 0.22) | -0.17  (-0.70 to 0.35) | -0.03  (-0.60 to 0.54) |
| CYP2D6  IM | **-0.67*****  **(-1.03 to -0.30)** | **-0.58****  **(-0.93 to -0.24)** | **-0.34***  **(-0.62 to -0.07)** | **-0.34***  **(-0.64 to -0.05)** |
| CYP2D6  NM | -0.00  (-0.32 to 0.32) | -0.00  (-0.28 to 0.28) | 0.05  (-0.19 to 0.30) | 0.05  (-0.22 to 0.31) |

**Significant values in bold text.** *p < 0.05, **p < 0.01, ***p < 0.001

Abbreviations: ABS_2D6_: anticholinergic burden score deriving from drugs metabolised by CYP2D6; CI: confidence interval; IM: intermediate metaboliser; MMSE: Mini-Mental State Examination; NM: normal metaboliser; PM: poor metaboliser

Model 1: unadjusted

Model 2: adjusted for anticholinergic burden score deriving from non-CYP2D6-metabolised drugs

Model 3: additionally adjusted for age and sex

Model 4: additionally adjusted for smoking status and depression

**Supplement 7:** Secondary analysis using quantile regression after exclusion of participants with concurrent use of CYP2D6 inhibitors and CYP2D6-metabolised anticholinergic drugs

|  | Model 1,  β (95% CI),  n = 857 | Model 2,  β (95% CI),  n = 857 | Model 3,  β (95% CI),  n = 857 | Model 4,  β (95% CI),  n = 816 |
| --- | --- | --- | --- | --- |
| *25^th^ percentile of MMSE score* | | | | |
| ABS_2D6_ | -0.00  (-0.44 to 0.44) | **-0.25***  **(-0.50 to -0.00)** | -0.01  (-0.30 to 0.28) | -0.01  (-0.32 to 0.31) |
| CYP2D6  PM | -0.20  (-1.32 to 0.92) | -0.24  (-1.45 to 0.98) | -0.22  (-1.37 to 0.93) | -0.04  (-0.76 to 0.68) |
| CYP2D6  IM | -0.00  (-0.79 to 0.79) | 0.00  (-0.57 to 0.57) | -0.25  (-0.76 to 0.26) | 0.03  (-0.49 to 0.55) |
| CYP2D6  NM | 0.00  (-0.68 to 0.68) | -0.00  (-0.30 to 0.30) | 0.03  (-0.34 to 0.40) | 0.04  (-0.39 to 0.46) |
| *50^th^ percentile of MMSE score* | | | | |
| ABS_2D6_ | **-0.33***  **(-0.61 to -0.06)** | **-0.25***  **(-0.50 to -0.00)** | -0.12  (-0.37 to 0.12) | -0.08  (-0.33 to 0.17) |
| CYP2D6  PM | -0.33  (-1.08 to 0.41) | -0.33  (-1.10 to 0.44) | -0.33  (-1.13 to 0.46) | -0.61  (-1.35 to 0.12) |
| CYP2D6  IM | **-0.50***  **(-0.91 to -0.09)** | **-0.50***  **(-0.93 to -0.07)** | -0.24  (-0.66 to 0.18) | -0.36  (-0.80 to 0.09) |
| CYP2D6  NM | 0.00  (-0.38 to 0.38) | 0.00  (-0.28 to 0.28) | 0.17  (-0.14 to 0.48) | 0.18  (-0.13 to 0.49) |
| *75^th^ percentile of MMSE score* | | | | |
| ABS_2D6_ | **-0.50*****  **(-0.73 to -0.27)** | **-0.33*****  **(-0.52 to -0.15)** | -0.01  (-0.20 to 0.17) | 0.00  (-0.19 to 0.19) |
| CYP2D6  PM | **-0.75***  **(-1.33 to -0.17)** | -0.50  (-1.20 to 0.20) | **-0.60***  **(-1.08 to -0.11)** | -0.10  (-0.57 to 0.38) |
| CYP2D6  IM | **-0.75*****  **(-1.18 to -0.32)** | **-0.58****  **(-0.96 to -0.21)** | **-0.47****  **(-0.77 to -0.17)** | **-0.50***  **(-0.82 to -0.18)** |
| CYP2D6  NM | -0.00  (-0.37 to 0.37) | -0.13  (-0.45 to 0.19) | 0.06  (-0.22 to 0.34) | 0.05  (-0.21 to 0.30) |

**Significant values in bold text.** *p < 0.05, **p < 0.01, ***p < 0.001

Abbreviations: ABS_2D6_: anticholinergic burden score deriving from drugs metabolised by CYP2D6; CI: confidence interval; IM: intermediate metaboliser; MMSE: Mini-Mental State Examination; NM: normal metaboliser; PM: poor metaboliser

Models 1-4 are defined as in Supplement 6.

**Supplement 8:** Quantile regression models for the association of anticholinergic burden deriving from drugs metabolised by CYP2D6 with MMSE score, grouping low-activity versus non-low-activity CYP2D6 phenotypes

|  | Model 1,  β (95% CI),  n = 872 | Model 2,  β (95% CI),  n = 872 | Model 3,  β (95% CI),  n = 872 | Model 4,  β (95% CI),  n = 830 |
| --- | --- | --- | --- | --- |
| *25^th^ percentile of MMSE score* | | | | |
| ABS_2D6_ | -0.00  (-0.39 to 0.39) | -0.06  (-0.42 to 0.30) | -0.05  (-0.32 to 0.21) | -0.00  (-0.28 to 0.28) |
| CYP2D6  PM + IM | -0.00  (-0.63 to 0.63) | -0.00  (-0.64 to 0.64) | -0.11  (-0.53 to 0.31) | -0.00  (-0.45 to 0.44) |
| CYP2D6  NM + UM | 0.00  (-0.63 to 0.63) | -0.00  (-0.31 to 0.31) | 0.03  (-0.32 to 0.38) | 0.04  (-0.36 to 0.43) |
| *50^th^ percentile of MMSE score* | | | | |
| ABS_2D6_ | **-0.33****  **(-0.58 to -0.08)** | **-0.33****  **(-0.56 to -0.11)** | -0.21  (-0.43 to 0.02) | -0.16  (-0.39 to 0.06) |
| CYP2D6  PM + IM | **-0.60*****  **(-0.92 to -0.28)** | **-0.33***  **(-0.66 to -0.00)** | -0.34  (-0.68 to 0.00) | -0.24  (-0.59 to 0.10) |
| CYP2D6  NM + UM | **-0.33***  **(-0.66 to -0.00)** | -0.08  (-0.35 to 0.18) | 0.02  (-0.26 to 0.30) | 0.06  (-0.22 to 0.34) |
| *75^th^ percentile of MMSE score* | | | | |
| ABS_2D6_ | **-0.50*****  **(-0.71 to -0.29)** | **-0.33*****  **(-0.51 to -0.16)** | -0.08  (-0.24 to 0.09) | -0.04  (-0.21 to 0.14) |
| CYP2D6  PM + IM | **-0.67*****  **(-0.96 to -0.37)** | **-0.58*****  **(-0.87 to -0.30)** | **-0.30***  **(-0.53 to -0.07)** | **-0.47*****  **(-0.72 to -0.21)** |
| CYP2D6  NM + UM | -0.00  (-0.31 to 0.31) | -0.20  (-0.48 to 0.08) | 0.03  (-0.21 to 0.28) | 0.04  (-0.23 to 0.30) |

**Significant values in bold text.** *p < 0.05, **p < 0.01, ***p < 0.001

Abbreviations: ABS_2D6_: anticholinergic burden score deriving from drugs metabolised by CYP2D6; CI: confidence interval; IM: intermediate metaboliser; MMSE: Mini-Mental State Examination; NM: normal metaboliser; PM: poor metaboliser

Models 1-4 are defined as in Supplement 6.

**REFERENCES**

1	Salahudeen MS, Duffull SB, Nishtala PS (2015) Anticholinergic burden quantified by anticholinergic risk scales and adverse outcomes in older people: a systematic review. BMC Geriatr 15:31. https://doi.org/10.1186/s12877-015-0029-9

2	FDA (2023) Drug Development and Drug Interactions: Table of Substrates, Inhibitors and Inducers. https://www.fda.gov/drugs/drug-interactions-labeling/drug-development-and-drug-interactions-table-substrates-inhibitors-and-inducers. Accessed February 13 2025

3	Flockhart DA (n.d.) Drug Interactions Flockhart Table™. https://drug-interactions.medicine.iu.edu/MainTable.aspx. Accessed February 13 2025
